# CIT-7, a crystalline, molecular sieve with pores bounded by 8 and 10-membered rings†
†Electronic supplementary information (ESI) available: Details of the synthesis and characterization of all materials as well as details on the synchrotron and RED data collection and structure determination, including the cif file. See DOI: 10.1039/c4sc03935a



**DOI:** 10.1039/c4sc03935a

**Published:** 2015-01-23

**Authors:** Joel E. Schmidt, Dan Xie, Thomas Rea, Mark E. Davis

**Affiliations:** a Chemical Engineering , California Institute of Technology , Pasadena , CA 91125 , USA . Email: mdavis@cheme.caltech.edu; b Chevron Energy Technology Company , Richmond , CA 94802 , USA

## Abstract

A new crystalline molecular sieve, denoted CIT-7, is synthesized using an imidazolium-based diquaternary organic structure directing agent (OSDA). The framework structure is determined from a combination of rotation electron diffraction and synchrotron X-ray powder diffraction data. The structure has 10 crystallographically unique tetrahedral atoms (T-atoms) in the unit cell, and can be described as an ordered arrangement of the [4^2^5^4^6^2^] *mtw* building unit and a previously unreported [4^4^5^2^] building unit. The framework contains a 2-dimensional pore system that is bounded by 10 T-atom rings (10-ring, 5.1 Å × 6.2 Å opening) that are connected with oval 8-rings (2.9 Å × 5.5 Å opening) through medium-sized cavities (∼7.9 Å) at the channel intersections. CIT-7 can be synthesized over a broad range of compositions including pure-silica and heteroatom, *e.g.*, aluminosilicate and titanosilicate, containing variants.

## Abstract 2

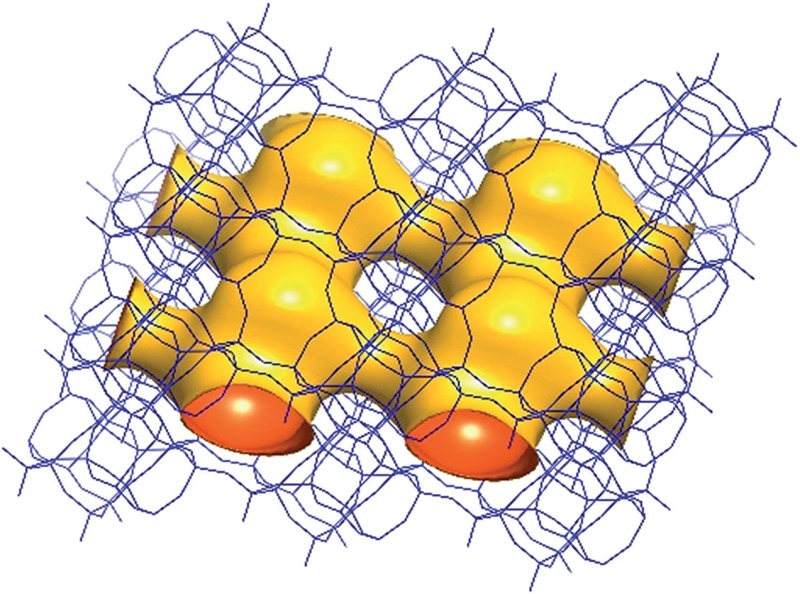
CIT-7, a new microporous material that contains 8 and 10-membered rings prepared as a silicate, an aluminosilicate or titanosilicate.

## 1. Introduction

The commercial utility of a molecular sieve arises from the combination of properties such as pore size, composition and hydrothermal stability that each structure can possess.^1–3^ The unique nature of each framework and properties often enables a single material to achieve optimal performance for a given application. Because of this phenomenon, there remains a strong motivation for creating new molecular sieve materials with new properties.^4^ One synthetic method that has been quite successful in the creation of new molecular sieves is the use of organic structure directing agents (OSDAs). Beginning with the pioneering work of Barrer and Denny,^5^ numerous mono-, di-, and polyquaternary OSDAs have been examined in the synthesis of microporous materials.^6–16^ The properties of OSDAs that show the greatest successes have been enumerated and the design of new OSDAs is now instrumental in the discovery of new molecular sieves; many have provided enhanced material properties, *e.g.*, catalytic activity and stability.

**Fig. 1:**
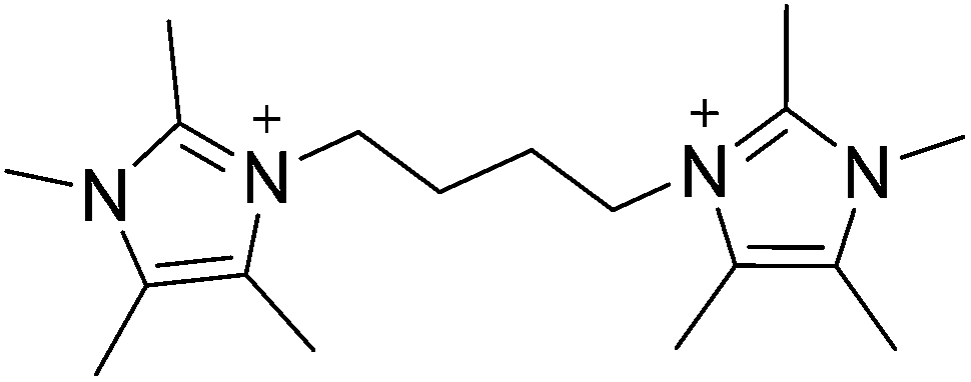
Diquaternary OSDA used to synthesize CIT-7.

Recently, we reported a computational method that was used to identify pentamethylimidazolium as being strongly directing towards pure-silica HPM-1 (**STW** framework type^17,18^).^19^ Based on the success of using pentamethylimidazolium, the simplest fully substituted imidazolium, we began with 1,2,4,5-tetramethylimidazole to prepare diquaternary (diquat) OSDAs. Here, we show that the diquat prepared using a 4-carbon chain length linker (Fig. 1) can be used to synthesize a new microporous material framework in pure-silica, fluoride mediated reactions. That same framework can be produced in aluminosilicate fluoride-mediated and hydroxide-mediated reactions with product compositions of Si/Al = 9–∞, as well as a titanosilicate material in fluoride-mediated syntheses. The structure of the new material, denoted CIT-7, is determined using a combination of synchrotron powder diffraction and rotation electron diffraction data.

## 2. Experimental

### 2.1. Organic structure directing agent

In a typical synthesis 200 mmol (24.8 g) of 1,2,4,5-teteramethylimidazole (TCI Chemicals) was dissolved in methanol. 100 mmol (21.6 g) of 1,4-dibromobutane (Sigma-Aldrich) was then added and the solution was refluxed overnight. The methanol was removed using rotary evaporation and the product was washed with ether to remove unreacted starting materials. The product was characterized using carbon NMR in D_2_O with methanol added as an internal standard, ^13^C-NMR (125 MHz, D_2_O): *δ* 7.83, 7.88, 9.67, 26.12, 31.48, 44.39, 124.81, 126.19, 142.10. HRMS-FAB (*m*/*z*): [M + H] calculated for C_18_H_31_N_4_, 303.25; found, 303.26. The product was converted from the iodide to the hydroxide form using hydroxide exchange resin (Dowex Marathon A) in water (all water used in these experiments was doubly-distilled deionized water from a MEGA-PURE® 6A Water Still) and the final concentration was determined using titration with a Mettler-Toledo DL22 autotitrator using 0.01 M HCl as the titrant.

### 2.2. Microporous materials synthesis

#### 2.2.1. Fluoride-mediated syntheses

A general synthesis procedure was as follows. First, tetraethylorthosilicate (Aldrich) was added to the OSDA in its hydroxide form. For aluminosilicates, aluminum isopropoxide (Aldrich) was then added and for titanosilicates titanium(iv) butoxide (Aldrich) was added. The container was closed, and stirred for at least 12 h to allow for complete hydrolysis. The lid was then removed, and the alcohol and appropriate amount of water were allowed to evaporate under a stream of air. Composition was monitored gravimetrically. Additional water was added as necessary, and then aqueous HF (48 wt%, Aldrich) was added and the mixture was stirred by hand until a homogenous gel was obtained. (Caution: use appropriate personal protective equipment, ventilation and other safety measures when working with HF.) If necessary, a second evaporation step was used after the addition of HF to reach the necessary water level. The final molar ratios are given in Table S1.† The autoclave was sealed and placed in a rotating oven at 175 °C. Aliquots of the material were taken periodically by first quenching the reactor in water and then removing enough material for X-ray powder diffraction (XPD).

#### 2.2.2. Hydroxide-mediated syntheses

The molar ratios used for hydroxide-mediated syntheses are given in Table S2.† In general, the OSDA in its hydroxide form, sodium hydroxide, any necessary water and sodium aluminate (Pfaltz & Bauer) were combined and stirred until the sodium aluminate completely dissolved. Ludox AS-40 (Aldrich) was then added and stirred until a homogenous gel was obtained. The gel pH was measured and then it was placed in a rotating oven at 160 °C. Aliquots were taken periodically and crystallization was monitored by both XPD and pH, as a jump in pH was generally observed when the product crystallized. After the product crystallized, the material was washed with water and then collected *via* centrifugation, this process was repeated at least three times and a final wash was performed using acetone. The product was dried at 100 °C in air.

For the synthesis using CBV 760 (dealuminated Y-zeolite with SiO_2_/Al_2_O_3_ = 60, Zeolyst International), 3 mmol of OSDA (based on charge) was mixed with 1 g of 1 M NaOH and water was added to bring the total mass to 7 g. Then 1 g of CBV 760 was added. The mixture was heated at 175 °C tumbling and monitored the same way as the other hydroxide reactions.

#### 2.2.3. Calcination

Products were calcined in breathing grade air. The material was heated to 150 °C at 1 °C min^–1^, held for three hours, then heated to 580 °C at 1 °C min^–1^ and held for six hours to assure complete combustion of the organic.

### 2.3. Characterizations

Liquid NMR spectra were recorded with a 500 MHz Spectrometer. The ^13^C CP-MAS NMR was recorded using a Bruker Avance 200 MHz spectrometer with a 7 mm rotor at a spinning rate of 4 kHz and were conducted in a 4.7 T magnetic field corresponding to Larmor frequencies of 200 MHz and 50.29 MHz for ^1^H and ^13^C respectively. The ^13^C NMR spectra are referenced to adamantane as a secondary external standard relative to tetramethylsilane. ^29^Si and ^19^F NMR were performed using a Bruker DSX-500 spectrometer (11.7 T) and a Bruker 4 mm MAS probe. The spectral frequencies were 500.2 MHz, 99.4 MHz, and 470.7 MHz for ^1^H, ^29^Si, and ^19^F nuclei, respectively, and spectra were referenced to external standards as follows: tetramethylsilane (TMS) for ^1^H and ^29^Si, and CFCl_3_ for ^19^F. The ^27^Al MAS NMR were recorded using a Bruker AM 300 MHz spectrometer with a 4 mm rotor at a spinning rate of 8 kHz, and were conducted in a 7.0 T magnetic field corresponding to a Larmor frequency of 78.172 MHz. The ^27^Al NMR spectra are referenced to 1.1 M Al(NO_3_)_3_ as an external standard.

Thermogravimetric analysis measurements were performed with a Netzsch STA 449C Jupiter. Samples were heated in air to 900 °C at a rate of 1 °C min^–1^.

Argon physical adsorption isotherms were performed at 87.45 K using a Quantachrome Autosorb iQ and were conducted using a quasi-equilibrium, volumetric technique.^20^

XPD data were collected on a Rigaku MiniFlex II with Cu Kα radiation.

Scanning electron micrographs (SEM) were recorded on a Hitachi S-570 instrument. EDS spectra were acquired with an Oxford X-Max SDD X-ray Energy Dispersive Spectrometer system on a ZEISS 1550 VP FESEM, equipped with in-lens SE.

Diffuse reflectance UV-visible (DRUV) spectra were recorded using a Cary 3G spectrophotometer equipped with a diffuse reflectance cell; zeolite samples were calcined using the method in Section 2.2.3 prior to data collection.

Details on the synchrotron XPD data collection can be found in the ESI,† Section 3.2. Three-dimensional electron diffraction data were collected on 2 crystals of CIT-7 using the rotation electron diffraction (RED) technique.^21,22^ The RED software was installed on a JEOL 2010 microscope operating at 200 kV, and data were collected over a tilt range of ±55° with a tilt step of 0.50° for the first set and 0.35° for the second set, the exposure time is 3 seconds per tilt step.

## 3. Results and discussion

### 3.1. Synthesis and characterization of pure-silica CIT-7

Complete experimental results for fluoride-mediated, pure-silica syntheses can be found in Table S1.† With H_2_O/SiO_2_ = 7, the product was pure-silica **STW**. This phase has already been reported using several different imidazolium based OSDAs.^19,23,24^ When the water contents of the reactions were decreased to H_2_O/SiO_2_ = 4, the OSDA led to the formation of a previously unknown phase. The XPD of the calcined material is shown in Fig. S1.† This material is denoted CIT-7 (California Institute of Technology number 7). Under these synthesis conditions, CIT-7 was found to crystallize along with **STW** as a competing phase, so care had to be taken to avoid the formation of **STW**. Once a pure-phase CIT-7 was obtained, seeds of CIT-7 were used in subsequent reactions to favor its formation over that of **STW**.

**Fig. 2:**
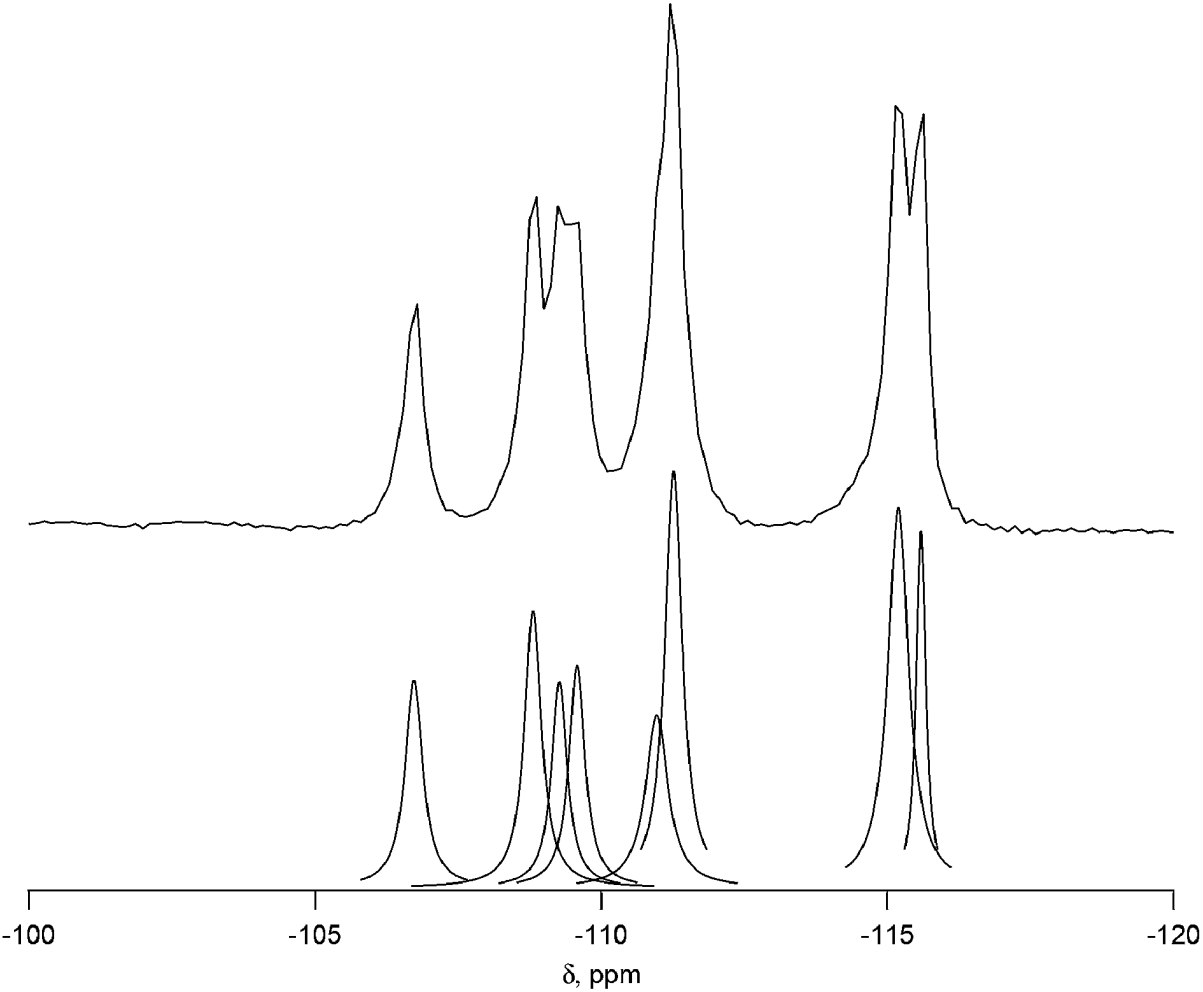
^29^Si NMR of calcined CIT-7 (upper) with the peak deconvolution (lower).

To demonstrate that the OSDA shown in Fig. 1, and not a decomposition product, formed CIT-7, the pure-silica material was analyzed with ^13^C CP-MAS NMR. The ^13^C CP-MAS NMR spectrum is compared to the liquid phase ^13^C NMR of the OSDA in Fig. S2,† and these data confirm that the OSDA is occluded intact in the material. The as-made, pure-silica material was stable to calcination in air at 580 °C to remove the OSDAs, and the TGA showed an organic content of 22.5 wt%. An argon adsorption isotherm of the calcined material is shown in Fig. S3,† and the micropore volume was found to be 0.19 cm^3^ g^–1^ (*t*-plot method, log plot of the data is provided in Fig. S4†). The ^19^F NMR of the as-made material is shown in Fig. S5† and reveals resonances at –45 ppm and –128 ppm. The resonance at –128 ppm can be assigned to a small amount of SiF_6_ in the sample, and the resonance at –45 ppm is consistent with fluoride being occluded in a pure-silica material.^25^ The ^29^Si NMR of the calcined material is shown in Fig. 2. All of the resonances in this material are assigned to Q^4^ silicon, that is Si(OSi)_4_ environments. The lack of any significant resonances in the Q^3^ region shows that this pure-silica framework has very low defects, and suggests that in the as-made material, the charge of the organic was compensated by occluded fluoride anions (shown to be present by ^19^F NMR).^25^

### 3.2. Structure determination of CIT-7

The structure of the calcined, pure-silica material was determined using a combination of synchrotron XPD and RED data. The calcined, pure-silica CIT-7 powder sample was packed into a 0.5 mm glass capillary and sealed. High-resolution XPD data were then collected on the 2-1 Powder Diffraction beamline at the Stanford Synchrotron Radiation Lightsource (SSRL).

The XPD pattern could be indexed with a triclinic unit cell (*a* = 13.020 Å, *b* = 11.205 Å, *c* = 9.375 Å, *α* = 92.8°, *β* = 107.2°, *γ* = 103.3°), using the program *TREOR* ^26^ implemented in the software *CMPR*.^27^ No indexing solutions on higher symmetry crystal systems could be found. Individual reflection intensities were extracted from the powder pattern to a minimum d-spacing of 0.90 Å (*ca.* 67.5° 2*θ*) using the program *EXTRACT* ^28^ in the *XRS-82* suite of programs.^29^ Structure solution using these data was then attempted using both the zeolite-specific structure-solution program *Focus*,^30^ and the powder charge-flipping algorithm^31^ in the program *Superflip*.^32^ Unfortunately, neither approach yielded a reasonable structural model.

Therefore, the RED technique^21,22^ was applied to the CIT-7 sample to obtain 3-dimensional single-crystal data (see ESI† Section 3.1). Two independent RED datasets were collected on two tiny crystallites, both could be indexed on triclinic unit cells that are similar to the one found for the XPD pattern. Reflection intensities (*ca.* 1.0 Å resolution) were then extracted for each data set using the RED software,^22^ and were further analyzed by the program *Triple*.^33^ Although both datasets gave data completeness of only *ca.* 55%, one did provide better quality over the other, *i.e.*, the agreement factor of the reflection intensities for Friedel pairs is 11.7% *versus* 22.1%. Therefore, a structure solution attempt using the better RED dataset for *Focus* structure solution (assuming the centro-symmetric space group *P*1) was performed. Many framework topologies were proposed by *Focus*, but none were chemically reasonable. Luckily, the two available RED datasets covered different areas of reciprocal space, and therefore by merging them, the data completeness could be improved to 86%. With the merged dataset included in the *Focus* runs, the structure solution became surprisingly straightforward. A model with 10 unique framework T-atoms, clearly showing a 2-dimensional channel system of intersecting 10- and 8-rings, was revealed. Indeed, this was the only solution proposed by the structure solution program.

**Fig. 3:**
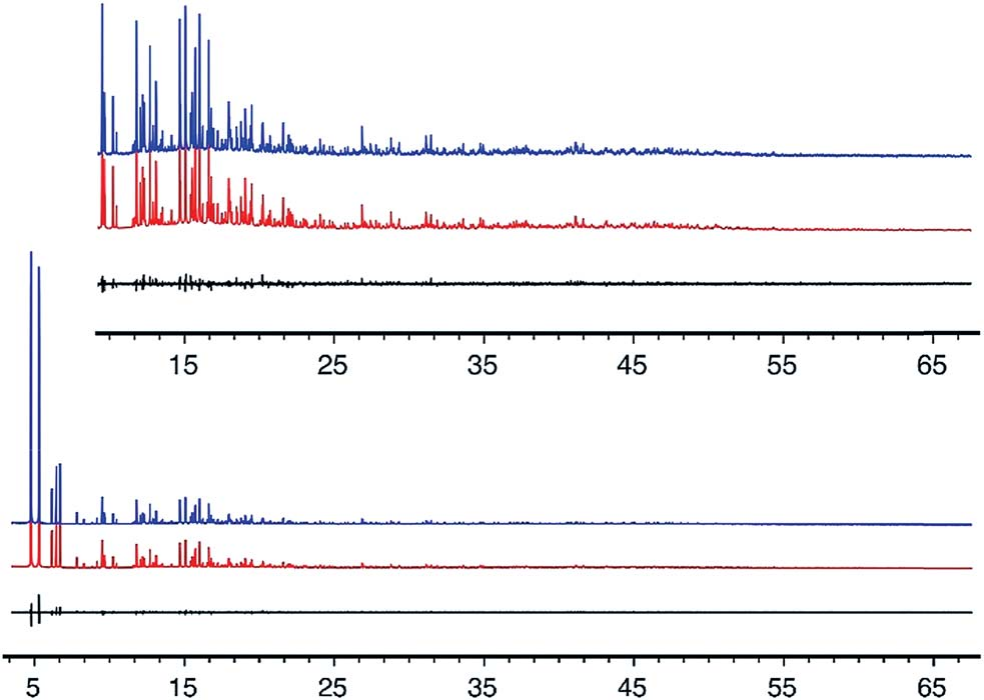
Observed (top), calculated (middle), and difference (bottom) profiles for the Rietveld refinement of pure-silica CIT-7. The profiles in the inset have been scaled up by a factor of 6 to show more detail.

The geometry of the CIT-7 framework structure model from the *Focus* run was optimized using the program *DLS-76*,^34^ and then served as a starting point for Rietveld refinement,^35^ using the synchrotron XPD data. Geometric restraints were applied on the bond distances and bond angles of the framework atoms, and their positions refined. These restraints were imposed throughout the refinement, but their relative weighting with respect to the XPD data was reduced as the refinement progressed. The structural model finally converged with *R*_F_ = 0.041 and *R*_wp_ = 0.077 (*R*_exp_ = 0.068). All atoms were refined isotropically using scattering factors for neutral atoms. The displacement parameters for similar atoms were constrained to be equal to keep the number of parameters to a minimum. Details of the refinement and selected bond distances and angles are given in Tables 1 and S3, and a cif file with the final atomic parameters is provided in the ESI.† The fit of the profile calculated from the final model to the experimental data is shown in Fig. 3.

**Table 1:** Crystallographic data for pure-silica CIT-7

Chemical composition	[Si_20_O_40_]
Unit cell	
*a* (Å)	13.0187(1)
*B* (Å)	11.2063(1)
*c* (Å)	9.3758(1)
*α* (°)	92.8224(6)
*β* (°)	107.2048(5)
*γ* (°)	103.2565(5)
Space group	*P*1
Number of observations	8001
Number of contributing reflections	3703
Number of geometric restraints	120
Number of structural parameters	98
Number of profile parameters	12
*R*_F_	0.041
*R*_wp_	0.077
*R*_exp_	0.068

Selected bond distances (Å) and angles (°)			
Si–O (Å)	Min: 1.59	Max: 1.62	Avg: 1.61
O–Si–O (°)	Min: 106.4	Max: 112.2	Avg: 109.5
Si–O–Si (°)	Min: 142.1	Max: 157.3	Avg: 148.9

### 3.3. Description and experimental support of CIT-7 structure

**Fig. 4:**
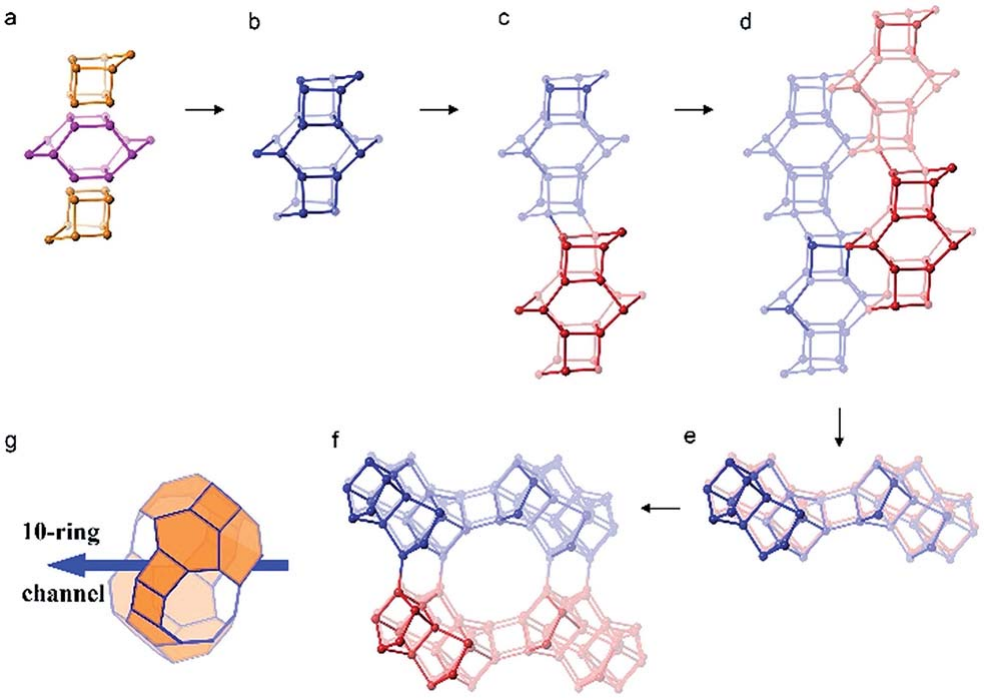
The construction of the framework structure CIT-7. (a) The *mtw* (in purple) and the new [4^4^5^2^] (in yellow) composite building units assembled to form (b) a repeating building block. (c) The connection of the building units in (b) to form a chain. (d) The arrangement of the chain (c) to form a layer with distorted 8-rings. (e) A different view of the layer in (d). (f) The connection of the layers in (d) to form 10-ring channels that are intersected with the 8-ring channels. (g) The [4^8^5^4^6^8^8^2^10^2^] cavity that can be accessed by 10-ring and 8-ring windows.

**Fig. 5:**
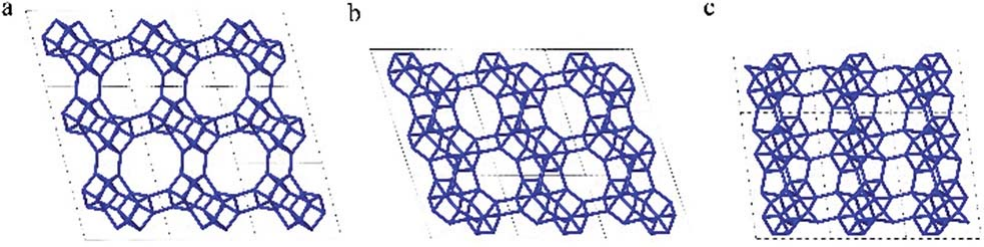
Framework structure of CIT-7. Projection view (3 × 3 × 3 unit cells) along the main crystallographic axis (a) [001], (b) [010], and (c) [100]. Framework O atoms have been omitted for clarity.

**Fig. 6:**
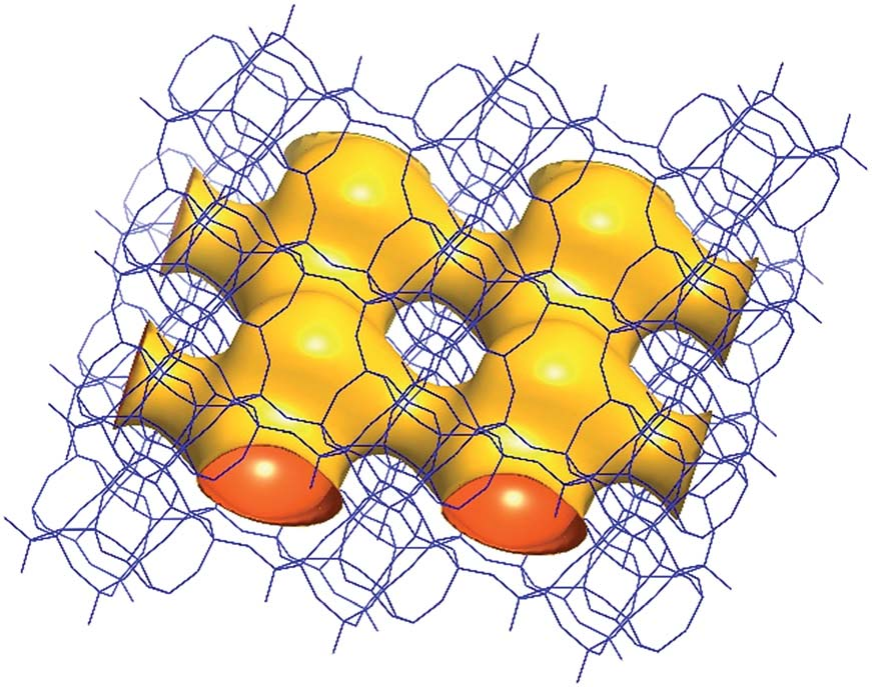
A structure envelope^44^ (yellow) highlighting the 10-/8-ring 2-dimensional channel system in the CIT-7 framework structure (blue).^45^

The framework structure of CIT-7 can be described as an ordered arrangement of a building block consisting of the [4^2^5^4^6^2^] *mtw* composite building unit (CBU), and a new [4^4^5^2^] building unit that has not been reported previously (*i.e*., it is not found in either the list of selected CBUs from the Database of Zeolite Structures^17^ or the exhaustive lists from Smith^36^ and van Koningsveld^37^) (Fig. 4a and b). The building block could be connected to the same neighbouring building block to form a chain (Fig. 4c) and the chain repeats itself to form a layer (Fig. 4d and e). As a result, oval 8-rings are created (2.9 Å × 5.5 Å opening, with the oxygen diameter of 2.70 Å subtracted). The layer that has 8-rings, could again link to itself and form 10-ring channels (5.1 Å × 6.2 Å opening, with the oxygen diameter of 2.70 Å subtracted) that are running perpendicular to the layer and intersected by 8-ring channels (Fig. 4f). At each intersection, a [4^8^5^4^6^8^8^2^10^2^] cavity is created (Fig. 4g). Alternatively, the CIT-7 framework structure could be described by natural tiling^38^ with a transitivity of [(10) (20) (16)4]. Four different types of tiles (*i.e*., [4^4^.5^2^], [5^2^.6^2^], [4^2^.5^4^.6^2^] and [4^8^.5^4^.6^8^.8^2^.10^2^]), derived by the program Topos,^39^ could be used to build the whole framework (see ESI† Section 4.1). The projection view along the three main crystallographic axes and the two-dimensional channel system are shown in Fig. 5 and 6, respectively.

It should be noted that the structure of CIT-7 is not predicted in any hypothetical zeolite framework databases as these databases only contain structures having less than 10 unique T atoms per unit cell. The optimized framework energy of pure-silica CIT-7 relative to α-quartz, *i.e*., 16.63 kJ mol^–1^ per Si atom, calculated by the program GULP^40^ that is implemented in the software Materials Studio 6.1,^41^ clearly demonstrates that this structure is energetically favorable.^42^ On the other hand, Li *et. al.* ^43^ recently proposed a set of criteria for evaluating chemical feasibility of zeolite frameworks, based on the statistics of the local interatomic distances (LIDs) rather than energy values. Calculations on the GULP-optimized CIT-7 structure indicated that this structure also fulfils all the LID criteria quite well.

The ^29^Si NMR spectrum of the calcined material is shown in Fig. 2 along with the deconvolution into 8 separate peaks. The chemical shifts, peak areas and T-site assignments are given in Table 2. The spectrum was deconvoluted into eight different resonances, with approximate area ratios of 1 : 2 : 2 : 1 : 1 : 1 : 1 : 1. The number of resonances and their area ratios are consistent with the structure solution having ten T-sites (there are ten unique T-sites in CIT-7, each with a site multiplicity of 2 as the space group is *P*1), and the chemical shifts and their area ratios correspond well with the average Si–O–Si bond angles. SEM images showing the morphology of the calcined, pure-silica material are provided in Fig. S6.† The observed low symmetry of the crystals is consistent with the low-symmetry found for the structure. Based on the structure solution, the TGA mass loss of 22.5 wt% corresponds well with 1 molecule of organic and 2 fluoride anions per unit cell. This organic content is also in agreement with the measured micropore volume.

**Table 2:** Chemical shifts from ^29^Si NMR of calcined pure silica CIT-7 along with the normalized peak areas and assigned T sites

Shift (ppm)	Normalized area	Assigned T site	Average Si–O–Si angle^*a*^
–115.6	0.92	Si10	152.2
–115.2	2.14	Si3 + Si8	151.7, 151.6
–111.3	1.97	Si1 + Si5	149.4, 149.1
–111.0	1.06	Si6	148.3
–109.6	0.97	Si9	147.2
–109.3	0.88	Si4	147.2
–108.8	1.28	Si7	146.8
–106.7	1.00	Si2	145.6

^*a*^From structure determination.

### 3.4. Heteroatom incorporation

#### 3.4.1. Aluminosilicate CIT-7

In fluoride-mediated reactions, CIT-7 was produced with gel compositions of Si/Al = 15, 25, 50, 100, 250 using seeded syntheses with pure-silica CIT-7 seeds (see ESI for synthesis details and results in Table S1 and a representative XPD in Fig. S7†). Of more importance to any practical application would be aluminosilicate materials produced in hydroxide mediated reactions. At Si/Al = 15 (see ESI for complete results in Table S2†), without the addition of seeds, **IWV** was found as the product (Fig. S8†). **IWV** is a 2-dimensional, 12-membered ring material that contains 14-membered rings that are only accessible through 12-membered rings. The aluminosilicate was first reported as ITQ-27, and was made using diphenyldimethylphosphonium as the OSDA. The synthesis is only reported at a difficult to achieve composition of: 1SiO_2_:0.014Al_2_O_3_:0.50Me_2_Ph_2_POH:0.50HF:4.2H_2_O, and takes 59 days to form, the addition of seeds only shortens this by one week.^46,47^

With the addition of pure-silica CIT-7 seeds to the aluminosilicate syntheses in hydroxide media, CIT-7 was produced instead of ITQ-27 (see XPD in Fig. S9†). Aluminosilicate CIT-7 could be easily obtained in hydroxide media at gel compositions of Si/Al = 5–15. Higher silica compositions led to products containing CIT-7 along with dense phases or ITQ-27. It is likely that optimizing these synthesis conditions will lead to higher silica products using a hydroxide mediated synthesis, however, these compositions are already accessible using the fluoride method. To demonstrate that the aluminum was in the framework, the calcined samples with the highest aluminum contents in both fluoride and hydroxide media were investigated by using ^27^Al NMR (Fig. S10†). In the sample prepared in hydroxide media, 95% of the aluminum was tetrahedral, and in the sample synthesized in fluoride media 88% was in tetrahedral coordination. In both of these samples, the majority of the aluminum was in tetrahedral coordination, demonstrating incorporation in the framework. The Si/Al range over which CIT-7 can be produced will allow for a wide variety of catalytic testing to be performed.

#### 3.4.2. Titanosilicate CIT-7

The ability of the CIT-7 framework to incorporate heteroatoms besides aluminum was tested by adding titanium to fluoride syntheses. Synthesis conditions are given in Table S1.† In the titanosilicate material, both Si/Ti = 50 and 100 were synthesized. DRUV of the as-made and calcined titanosilicate materials was used to show that the titanium was present in tetrahedral coordination (Fig. S11†), indicating framework incorporation.

### 3.5. Additional OSDAs

In addition to the OSDA shown in Fig. 1, we found that 2-ethyl-1,3-dimethylimidazolium can form CIT-7. However, this OSDA was not as strongly directing as the diquat as it was very difficult to produce a pure-phase sample, as the product was often contaminated with **ITW**, **MTW**, **STF** and **STW**. In our investigations of diquats of varying linker length using tetramethylimidazole, we have found that linkers of 3 and 5 carbons can also form CIT-7, but these are not as strongly directing towards this framework. Further studies on diquats of varying linker lengths are in progress.

### 3.6. Comparison to known materials

**Fig. 7:**
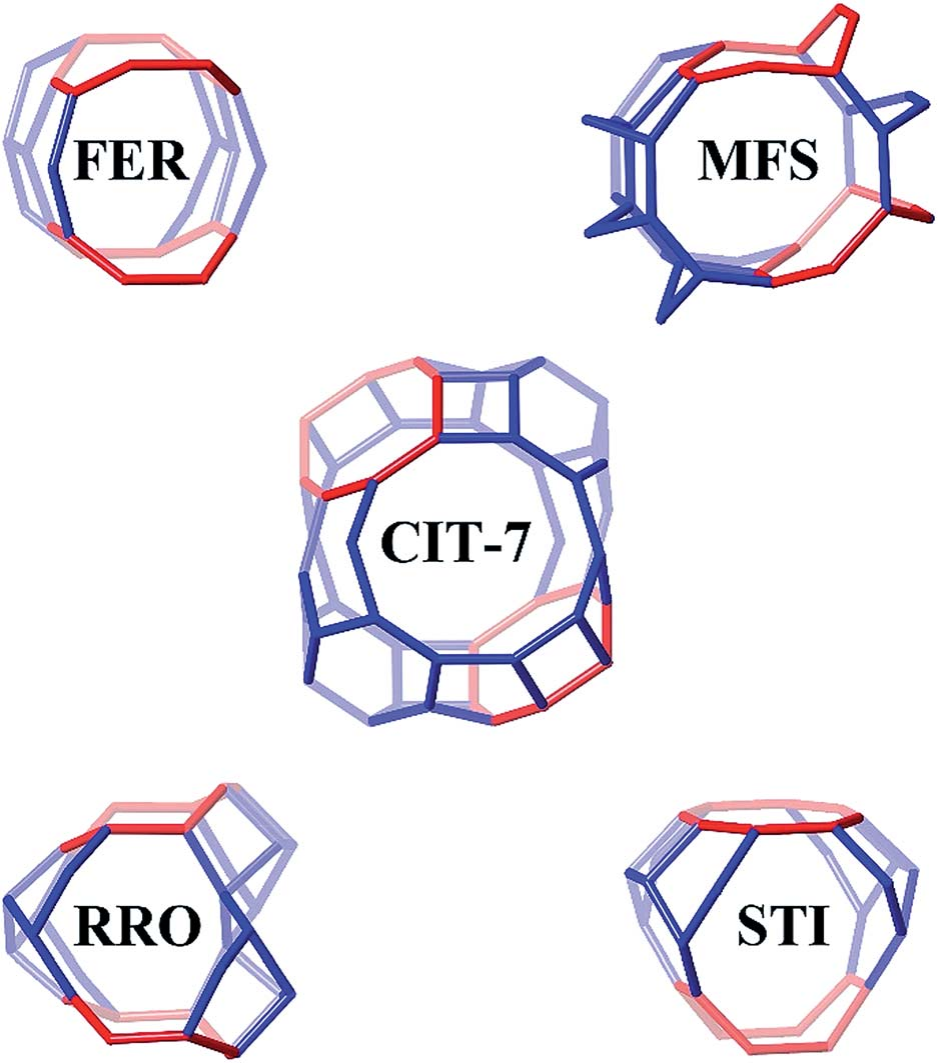
The 10-/8-ring channel intersections for CIT-7, **FER**, **MFS**, **RRO** and **STI**. The 8-rings are highlighted in red. Bridging O atoms have been omitted for clarity.

Four other zeolite frameworks, *i.e.*, **FER** (*e.g.*, NU-23, ZSM-35), **MFS** (*e.g.*, ZSM-57), **RRO** (*e.g.*, RUB-41) and **STI** (*e.g.*, TNU-10, SSZ-75), have a 2-dimensional 10-/8-ring channel systems. CIT-7 distinguishes itself from these known materials by some unique structural features. As shown in Fig. 7, CIT-7 is the only system that has a large cavity in the intersection region. The maximum included sphere diameter for the idealized CIT-7 framework (*i.e.*, the one after distance-angle least-square refinement^34^) is calculated to be 7.91 Å, significantly larger than those for the other four idealized frameworks (Table 3). Also, it should be noted that CIT-7 can be made across a very wide Si/Al ratio (9–∞) as well as a titanium (and we suspect other heteroatoms) containing material. This compositional flexibility, when combined with the medium-/small-pore channels and intersecting cavities, could be of interest in a broad spectrum of applications.

**Table 3:** Comparison of the channel and pore characteristics for the five 2D 10-/8-ring zeolites. For the 4 known zeolite frameworks, the channel characteristics are taken from the literature,^48^ and the pore characteristics are taken from the Database of Zeolite Structures.^17^ The channel characteristics for CIT-7 are calculated using the program “Sphere Viewer”.^49^ All data are in Å^*a*^

Framework	*D*_M_	*D*_*a*_	*D*_*b*_	*D*_*c*_	Material	10R opening	8R opening
Idealized CIT-7	7.91	1.87	2.92	4.67	CIT-7	5.1 × 6.2	2.9 × 5.5
**MFS**	6.71	5.31	3.14	1.51	ZSM-57	5.1 × 5.4	3.3 × 4.8
**FER**	6.25	1.50	3.34	4.63	Ferrierite	4.2 × 5.4	3.5 × 4.8
**STI**	6.23	4.88	2.90	1.79	SSZ-75	4.7 × 5.0	2.7 × 5.6
**RRO**	4.40	4.03	1.48	3.07	RUB-41	4.0 × 6.5	2.7 × 5.0

^*a*^Note: *D*_M_ means the maximum included sphere diameters, *D*_*a*_, *D*_*b*_ and *D*_*c*_ are the maximum free sphere diameters that can diffuse along *a*-, *b*- and *c*-axis, respectively.

Other materials with 2-dimensional 10-/8-ring channel systems have been proposed for applications including carbonylation, NO_X_ reduction, methanol-to-olefins, amine synthesis, and gas separations.^50–52^ Among these materials, it has been reported that ferrierite has been commercialized for isomerization of butenes and pentenes in the Isomplus process (Shell and Lyondell Petrochemical) on a 10,000 BPD butene feed at the Equistar Chemicals facility in Channelview, TX.^2,53^ A modified ferrierite is also used as a catalyst in olefin skeletal isomerization in the Isotex process by Texaco.^53–55^ These examples illustrate the potential utility of 2-dimensional 10-/8-ring channel systems. Thus, it will be interesting to compare CIT-7 to these known materials, and we are currently in progress of testing them in a number of catalytic reactions.

## 4. Conclusions

We have reported here the synthesis, structural solution and characterization of a new microporous material with a 10-/8-ring, 2-dimensional channel system, with large cages at the intersections. This material can be prepared across a wide range of compositions and exhibits good stability. As microporous materials with a similar 2-dimensional 10-/8-ring channel systems have already been commercialized, we are currently investigating the catalytic applications of CIT-7 to find the influence of the unique features of this framework, such as the large cage at channel intersections.
