# Supplementary material for: CIT-7, a crystalline, molecular sieve with pores bounded by 8 and 10-membered rings
Source: Chem Sci. 2015 Jan 23;6(3):1728–34. doi: 10.1039/c4sc03935a (PMC5643978; doi:10.1039/c4sc03935a)
Supplement: Supplementary file 1 [file SC-006-C4SC03935A-s001.pdf]

Electronic Supplementary Material (ESI) for Chemical Science.
This journal is © The Royal Society of Chemistry 2015

CIT-7, a Crystalline, Molecular Sieve with Pores Bounded by 8 and 10-Membered Rings

Joel E. Schmidt[1], Dan Xie[2], Thomas Rea[2] and Mark E. Davis[1]*

[1]Chemical Engineering, California Institute of Technology, Pasadena, CA 91125

[2]Chevron Energy Technology Company, Richmond, CA 94802, USA

*mdavis@cheme.caltech.edu

Supporting Information

1. Microporous Material Synthesis Results

Table S1. Fluoride mediated synthesis results.

Gel Ratios							Product Ratios[b]	
Si/Al	Si/Ti	H_2O/SiO_2	Seeds	Temp (°C)	Time (days)	Result	Si/Al	Si/Ti
∞	-	4	None	175	8	STW[a]	-	-
∞	-	4	None	175	6	STW+CIT-7[a]	-	-
∞	-	4	None	175	6	CIT-7[a]	-	-
∞	-	4	Silica CIT-7	175	6	CIT-7	-	-
∞	-	7	None	175	6	STW	-	-
15	-	4	Silica CIT-7	175	5	CIT-7	10	-
20	-	4	None	175	20	CIT-7	14	-
20	-	4	None	175	12	CIT-7	13	-
25	-	4	Silica CIT-7	175	5	CIT-7	15	-
25	-	4	Silica CIT-7	175	5	CIT-7	17	-
25	-	4	Silica CIT-7	175	6	CIT-7	14	-
50	-	4	None	175	18	CIT-7	27	-
50	-	4	Silica CIT-7	175	4	CIT-7	28	-
100	-	4	Silica CIT-7	175	4	CIT-7	36	-
250	-	4	Silica CIT-7	175	4	CIT-7	225	-
-	50	4	Silica CIT-7	175	7	CIT-7	-	63
-	100	4	Silica CIT-7	175	7	CIT-7	-	88

[a]Since STW and CIT-7 were competing products some syntheses produced pure phase versions (per XPD) of those molecular sieves
[b]Determined using EDS of calcined material

Table S2. Hydroxide mediated synthesis results.

Gel Si/Al	Gel Na/Si	Gel ROH/Si	Gel H_2O/Si	Temp (°C)	Seeds	Time (days)	Product	Product Si/Al
5[a]	0.25	0.16	30	160	Silica CIT-7	35	CIT-7	9
10[a]	0.25	0.16	30	160	None	20	CIT-7	12
15[a]	0.16	0.16	30	160	None	35	IWV	
15[a]	0.16	0.16	30	160	Silica CIT-7	10	CIT-7	18.4 H^+ form
15[a]	0.16	0.16	30	160	Silica CIT-7	10	CIT-7	9
30[b]				175	None	18	IWV	29
30[b]				175	Silica CIT-7	23	IWV+CIT-7	

[a]Made using Ludox AS-40 and sodium aluminate
[b]Made from CBV760

2. Characterization

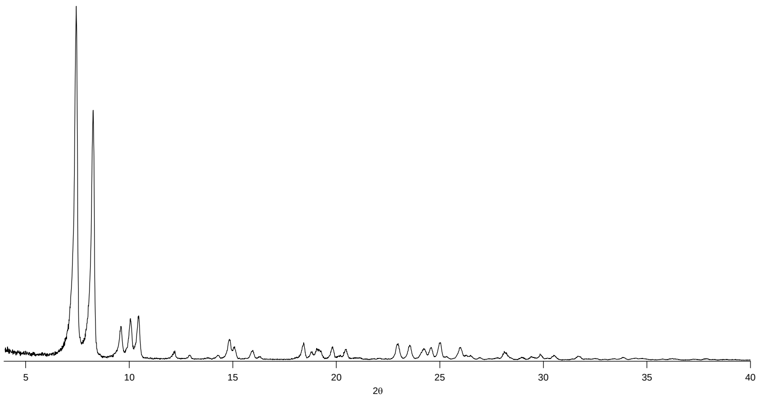

Figure S1. XPD pattern of calcined pure-silica CIT-7 produced in fluoride media.

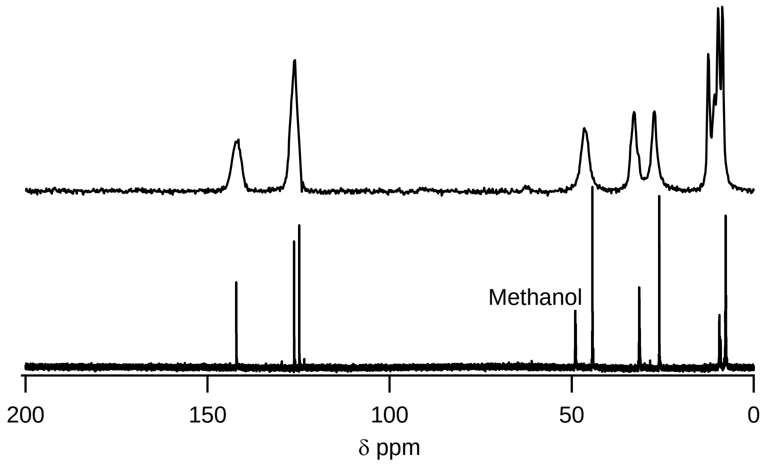

Figure S2. ^{13}C CP-MAS NMR of as-made CIT-7 (upper) showing the occluded OSDA and comparison to the liquid NMR (lower, methanol added as an internal standard).

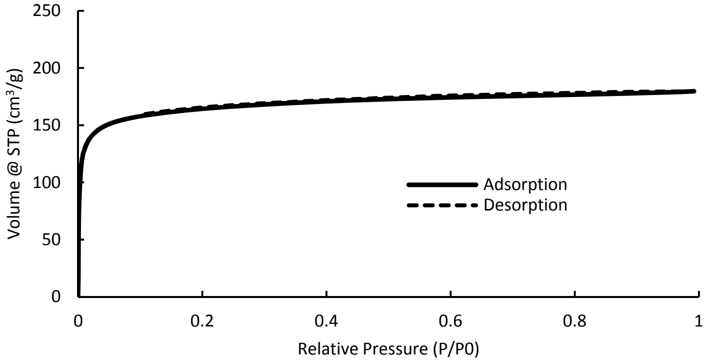

Figure S3. Argon isotherm of CIT-7.

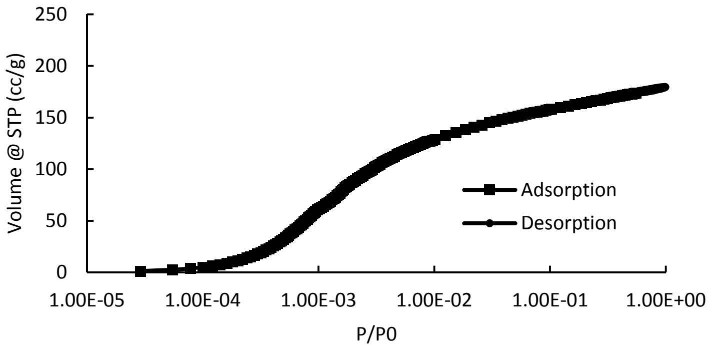

Figure S4. Log plot argon adsorption isotherm.

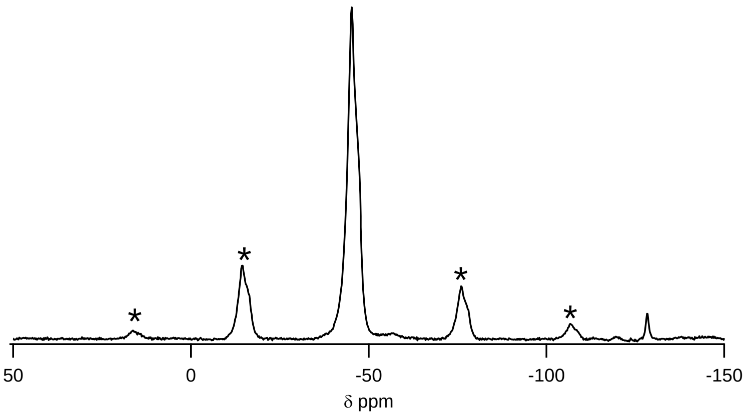

Figure S5. ^{19}F NMR of as-made CIT-7, spinning side bands are marked with an asterisk.

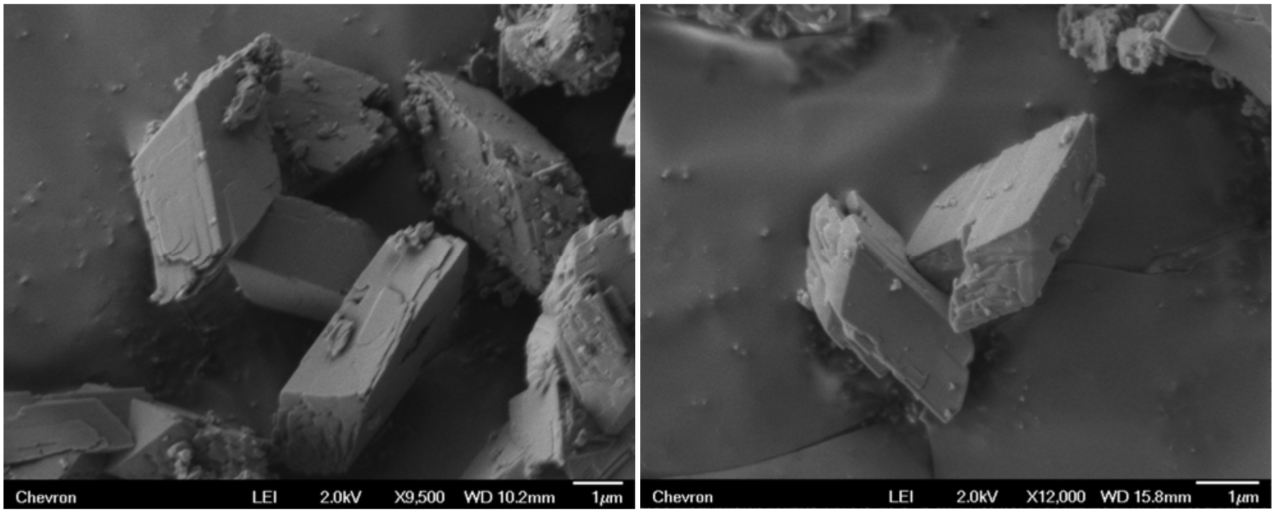

Figure S6. SEM images of calcined, pure-silica CIT-7.

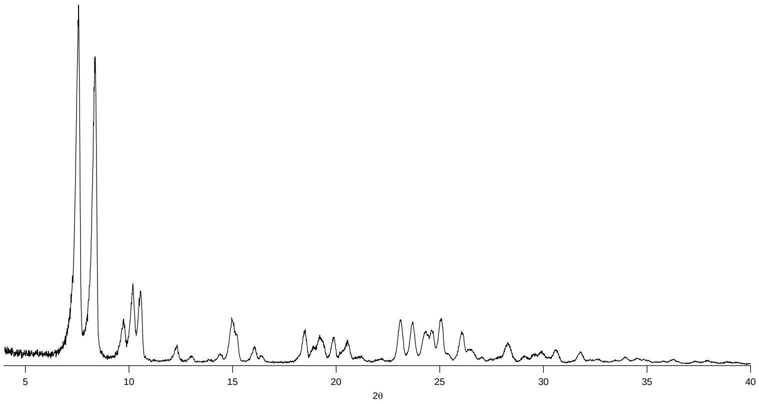

Figure S7. XPD pattern of calcined aluminosilicate CIT-7 produced in fluoride media with gel Si/Al=50.

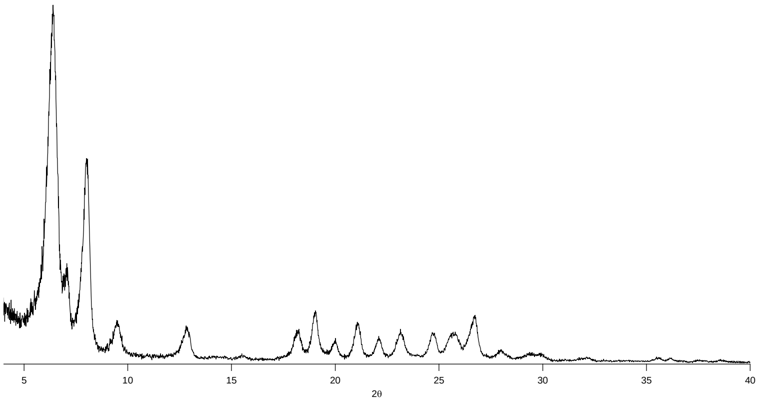

Figure S8. XPD pattern of calcined ITQ-27.

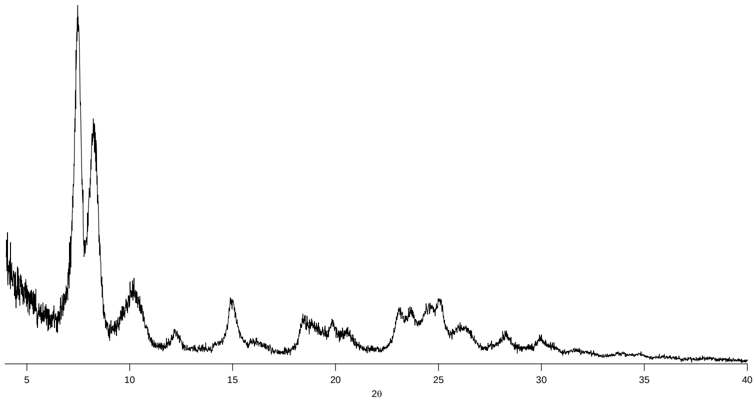

Figure S9. XPD pattern of as-made CIT-7 produced in hydroxide media with gel Si/Al=15.

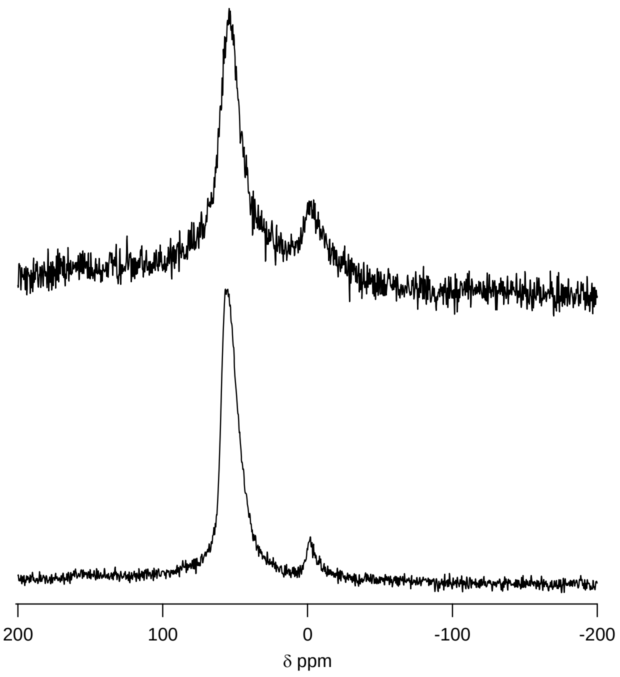

Figure S10. ^{27}Al MAS NMR of aluminosilicate CIT-7. Upper is fluoride mediated synthesis with gel Si/Al=15 and lower is hydroxide mediated synthesis with gel Si/Al=5. The sample made in hydroxide media is 95% tetrahedral aluminum and the sample made in fluoride media is 88% tetrahedral aluminum.

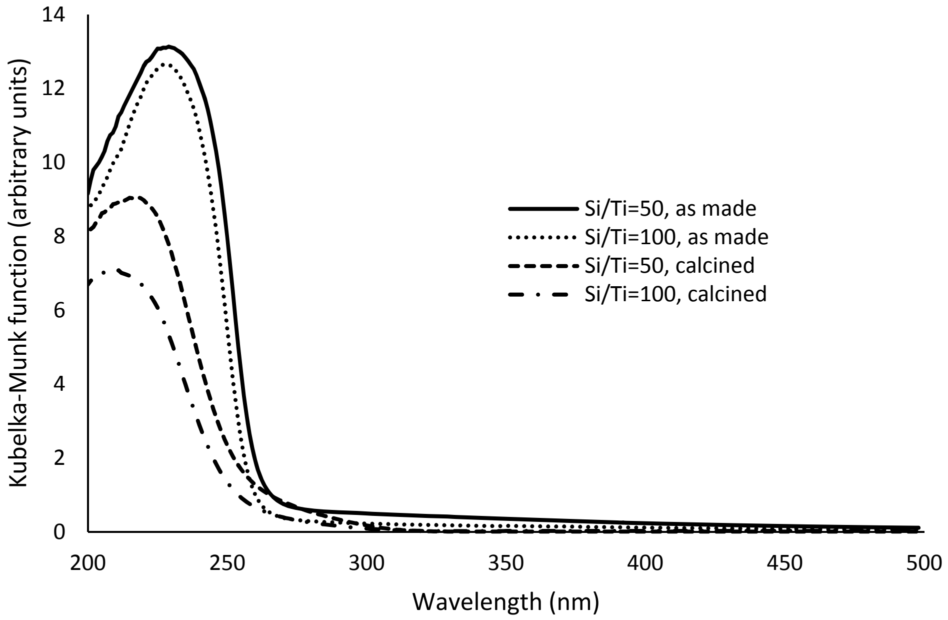

Figure S11. UV-VIS of titanosilicate CIT-7.

3. Structure Determination

3.1 Rotation Electron Diffraction Data Collection

Large tilt steps (0.5°/0.35°) had to be used for these measurements, because the RED software to perform the finer tilts by tilting the electron beam had not yet been implemented to the JEOL 2010 TEM. As a result, the RED data were not of optimal quality.

Table S3. RED data collection.

	Dataset 1	Dataset 2	Merged dataset
Tilt range (in °)	-55 -> +60	-55 -> +60	/
Tilt step size (in °)	0.50	0.35	/
Number of 2-dimensional ED images	262	296	/
Collected reflections	2312	2248	3590
Independent reflections	1315	1289	2007
Data resolution (in Å)	1.0	1.0	1.0
Agreement factor of the reflection intensities for Friedel pairs	11.7%	22.1%	16.8%
Data completeness	56.0%	54.9%	85.5%

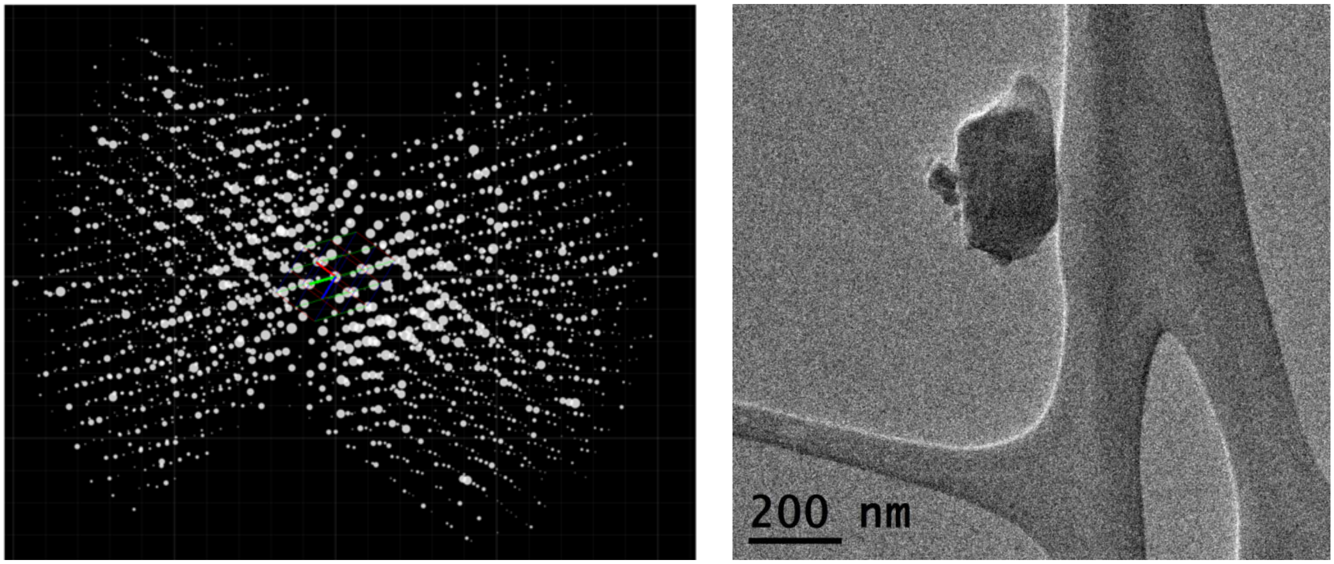

Figure S12. 3D electron diffraction tomography data (left) collected from a calcined, pure-silica CIT-7 (right).

3.2 Synchrotron XPD Data Collection.

Table S4. Synchrotron XPD data collection.

Synchrotron facility	2-1 Beamline at SSRL
Wavelength	0.99995 Å
Diffraction geometry	Debye-Scherrer
Analyzer crystal	Si 1 1 1
Sample	Rotating 0.5 mm capillary
2θ range	3.5-73.5°
Step size	0.004°2θ
Time per step	
3.5-5.8°2θ	2.0 s
5.8-19.8°2θ	4.0 s
19.8-73.5°2θ	6.0 s

4. Description of The CIT-7 Framework Structure

4.1 Natural Tiling Analysis of the CIT-7 Framework

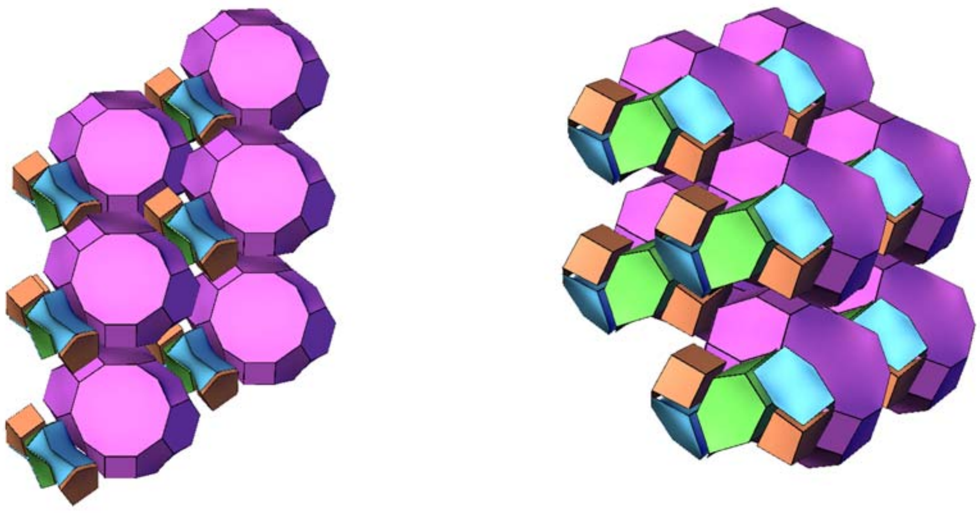

Figure S13. Tiling of the CIT-7 framework built by 4 different types of tiles [$5^2.6^2$] (blue), [$4^4.5^2$] (Orange), [$4^2.5^4.6^2$] (green) and [$4^8.5^4.6^8.8^2.10^2$] (purple), viewing down the [011] projection (left) and the [111] projection (right). The software TOPOS [S1] was used to analyze the framework topology, and the software 3dt [S2] was used for visualizing the tiles.

Transitivity: [(10)(20)(16)4]; Natural Tiling Signature: 2[$5^2.6^2$]+2[$4^4.5^2$]+[$4^2.5^4.6^2$]+[$4^8.5^4.6^8.8^2.10^2$]

Coordination Sequences:

Si1: 4 12 17 27 49 79 99 120 146 192	Si2: 4 9 18 30 49 73 100 117 150 203
Si3: 4 10 18 31 47 75 98 124 156 189	Si4: 4 10 19 32 55 70 89 124 160 199
Si5: 4 12 20 29 49 73 100 129 157 186	Si6: 4 10 21 35 46 65 99 134 162 191
Si7: 4 9 19 37 50 66 92 126 164 200	Si8: 4 10 18 30 48 71 105 126 145 193
Si9: 4 9 18 32 48 69 95 131 160 182	Si10: 4 11 19 30 45 73 102 125 156 187

4.2 The crystallographic information file (cif) for the pure-silica CIT-7.

```
data_all-silica_CIT-7
_chemical_name_systematic
"CIT-7"
_chemical_formula_structural
"[Si20O40]"

_cell_length_a          13.0187(1)
_cell_length_b          11.2063(1)
_cell_length_c           9.3758(1)
_cell_angle_alpha       92.8224(6)
_cell_angle_beta       107.2048(5)
_cell_angle_gamma      103.2565(5)

_symmetry_space_group_name_H-M    'P -1'
_symmetry_Int_Tables_number       2
_symmetry_cell_setting          triclinic
loop_
_symmetry_equiv_pos_as_xyz
'+x,+y,+z'
'-x,-y,-z'

loop_
_atom_site_label
_atom_site_type_symbol
_atom_site_occupancy
_atom_site_fract_x
_atom_site_fract_y
_atom_site_fract_z
_atom_site_U_iso_or_equiv
   O1   O 1.0000    0.2236(2)  0.5059(3)  0.0497(2)  1.07(10)
   O2   O 1.0000    0.3564(1)  0.4201(2)  0.2714(5)  1.07
   O3   O 1.0000    0.3240(2)  0.6411(2)  0.3146(3)  1.07
   O4   O 1.0000    0.1662(1)  0.4463(4)  0.2860(1)  1.07
   O5   O 1.0000    0.3703(1)  0.6646(2)  0.9715(3)  1.07
   O6   O 1.0000    0.1691(1)  0.5937(2)  0.7893(2)  1.07
   O7   O 1.0000    0.2986(1)  0.4377(2)  0.8372(3)  1.07
   O8   O 1.0000    0.3411(3)  0.2514(1)  0.7003(3)  1.07
   O9   O 1.0000    0.4275(3)  0.4816(3)  0.6650(3)  1.07
  O10   O 1.0000    0.2112(2)  0.3738(3)  0.5487(3)  1.07
  O11   O 1.0000    0.1312(2)  0.5669(1)  0.4991(2)  1.07
  O12   O 1.0000    1.0012(2)  0.3462(3)  0.3897(2)  1.07
  O13   O 1.0000    0.3314(3)  0.0139(1)  0.6712(3)  1.07
  O14   O 1.0000    0.5093(1)  0.1725(3)  0.6611(2)  1.07
  O15   O 1.0000    0.4571(2)  0.1536(2)  0.9168(3)  1.07
  O16   O 1.0000    0.4093(5)  0.8471(2)  0.8129(3)  1.07
  O17   O 1.0000    0.2014(1)  0.7903(2)  0.6425(2)  1.07
  O18   O 1.0000    0.3636(1)  0.8314(3)  0.5162(2)  1.07
  O19   O 1.0000    0.4693(2)  0.3106(2)  0.1474(2)  1.07
  O20   O 1.0000    0.4992(2)  0.3135(2)  0.4408(2)  1.07
  Si1   Si 1.0000   0.2680(1)  0.5033(1)  0.2286(2)  0.80(5)
  Si2   Si 1.0000   0.2646(1)  0.5496(2)  0.9108(1)  0.80
  Si3   Si 1.0000   0.3210(2)  0.3874(1)  0.6886(2)  0.80
  Si4   Si 1.0000   0.1261(1)  0.4313(1)  0.4305(2)  0.80
  Si5   Si 1.0000   0.4105(2)  0.1493(2)  0.7367(1)  0.80
  Si6   Si 1.0000   0.3255(1)  0.8692(2)  0.6586(2)  0.80
  Si7   Si 1.0000   0.1261(1)  0.6529(1)  0.6366(2)  0.80
  Si8   Si 1.0000   0.4754(2)  0.3911(2)  0.2987(2)  0.80
  Si9   Si 1.0000   0.4638(2)  0.7620(1)  0.9296(2)  0.80
 Si10   Si 1.0000   0.4203(1)  0.7469(2)  0.4335(2)  0.80
```

5. References

[S1] V. A. Blatov, O. Delgado-Friedrichs, M. O'Keeffe, D. M. Proserpio, *Acta Cryst. A.*, 2007, **63**, 418–425.
[S2] O. Delgado-Friedrichs, M. O’Keeffe, *Acta Cryst. A.*, 2003, **59**, 351-360.
